# Supplementary material for: Non-canonical Wnt signalling regulates scarring in biliary disease via the planar cell polarity receptors
Source: Nat Commun. 2020 Jan 23;11:445. doi: 10.1038/s41467-020-14283-3 (PMC6978415; doi:10.1038/s41467-020-14283-3)
Supplement: Supplementary file 1 — Supplementary Information [file 41467_2020_14283_MOESM1_ESM.pdf]

Wilson et al. Non-canonical Wnt signalling regulates scarring in biliary disease via planar cell polarity receptors

Supplementary Discussion:

Wnt-β-catenin activity does not increase during BEC proliferation: BEC proliferation is required during bile duct regeneration[1]; however, the role that Wnt-β-catenin signalling plays in this process remains controversial, with conflicting reports describing variable roles for Wnt-β-catenin[2–5].

In many cellular contexts, *Axin2* mRNA expression is an accepted readout of Wnt-β-catenin signalling pathway activation[6–9]. Similarly, in both mouse and human BECs, *Axin2* mRNA levels increases when β-catenin is stabilised using a selective GSK3β inhibitor, CHIR99021, and decreases when β-catenin-dependant transcription is inhibited by PRI724[10] (Supplementary Figure 1a), indicating that BECs respond to activation of the Wnt-β-catenin signalling pathway by altering *Axin2* transcript expression. Using a combination of β-catenin nuclear translocation and *Axin2* mRNA expression, we sought then to define whether Wnt-β-catenin signalling is active in proliferating BECs *in vivo*.

As previously reported, in mice expressing a stabilising β-catenin^{N90} mutation in the liver[11,12] or in the mouse small intestine[13], β-catenin protein localises to the nucleus of cells identifying them as having an activated Wnt-β-catenin signalling pathway (Supplementary Figure 1b). In tissue from patients with Primary Sclerosing Cholangitis (PSC), a progressive human biliary disease in which BECs proliferate[14] and also in two mouse models of BEC proliferation (thioacetamide, TAA or 3,5-diethoxycarbonyl-1,4-dihydrocollidine, DDC[15,16]), we failed to see β-catenin translocate into the nucleus of BECs (Supplementary Figure 1c and 1d). Given that the nuclear epitope of endogenous β-catenin is difficult to detect, we validated our results using RNAScope for *Axin2*. In both mouse models of BEC proliferation, *Axin2* transcripts are localised to peri-central hepatocytes where Wnt-β-catenin signalling occurs[17]; however, in both uninjured and proliferating BECs *Axin2* mRNAs remain infrequent (Supplementary Figure 1d and 1e). Finally, we verified this by isolating healthy murine bile ducts and ducts from mice with biliary disease (Supplementary Figure 1f). In this context, we did not see an increase in *Axin2* mRNA levels, nor did we see an increase in expression of other known Wnt- β-catenin target genes (supplementary figure 1g). We cannot discount that there is a low, tonic level of Wnt-β-

catenin signalling in proliferating BECs; however, our data indicate that Wnt-β-catenin pathway activation does not increase during bile duct regeneration, data that are in concordance with recent work showing that BECs do not express LGR-proteins necessary for Wnt signalling potentiation[3,7]. Moreover, recent functional evidence suggests that the canonical Wnt signalling pathway is dispensable for BEC organoid growth in vitro[18].

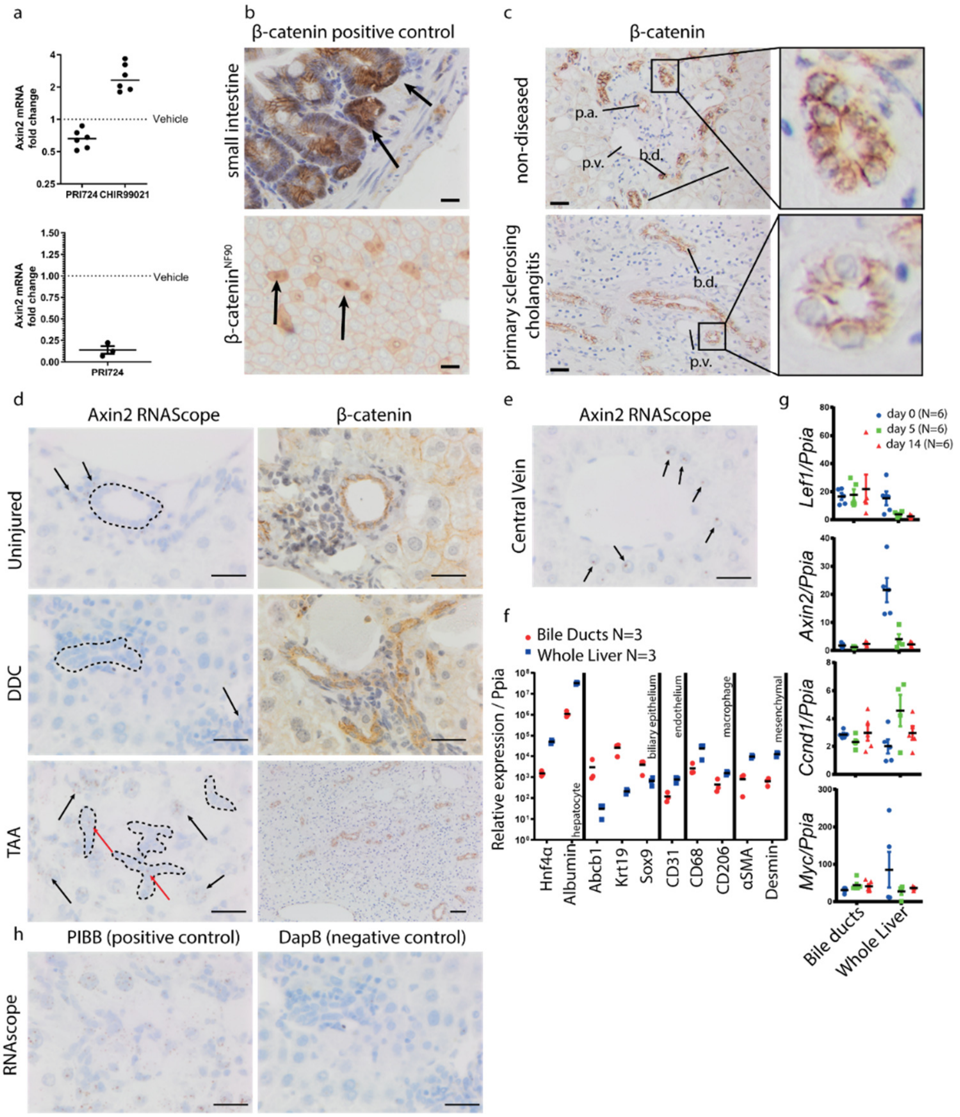

Supplementary Figure 1. Limited evidence for canonical Wnt signalling during bile duct regeneration: a. mRNA expression of *Axin2* in NHC3 human cholangiocytes (upper graph) treated with PRI724 or CHIR099021 and primary mouse cholangiocyte organoids (lower graph) treated with PRI724. Each point represents an experimental replicate. **b.** Immunohistochemistry for β-catenin in positive control tissue, small intestine (upper panel) and liver expressing β-catenin^{NF90}. **c.** Immunohistochemistry for β-catenin in healthy human liver and tissue from a PSC patient. **d.** RNAScope for *Axin2* in uninjured mouse liver and in mice with DDC or TAA induced liver injury. Black arrows denote positive staining in hepatocytes, red arrows demarcate *Axin2* positivity in biliary structures. Dotted line denotes bile ducts **e.** *Axin2* mRNA positive control, black arrows denote *Axin2* positive pericentral hepatocytes. **f.** mRNA expression of lineage markers for hepatocytes, biliary epithelium, endothelium, macrophages and mesenchymal cells. **g.** mRNA expression of canonical Wnt target genes in whole liver and in isolated bile ducts from the DDC bile duct injury model. **h.** RNA scope for PIBB and DapB, positive and negative RNAscope controls. Scale bar = 50µm p.a. denotes portal artery, p.v. denotes portal vein and b.d. denotes bile duct. Source data are provided as a Source Data file. In dot-plots, data presented as mean ± S.E.M. Each data-point (N) represents an individual animal. In supplementary 1a, N=6 experimental replicates for the upper panel and N=3 experimental replicates for the lower panel.

a

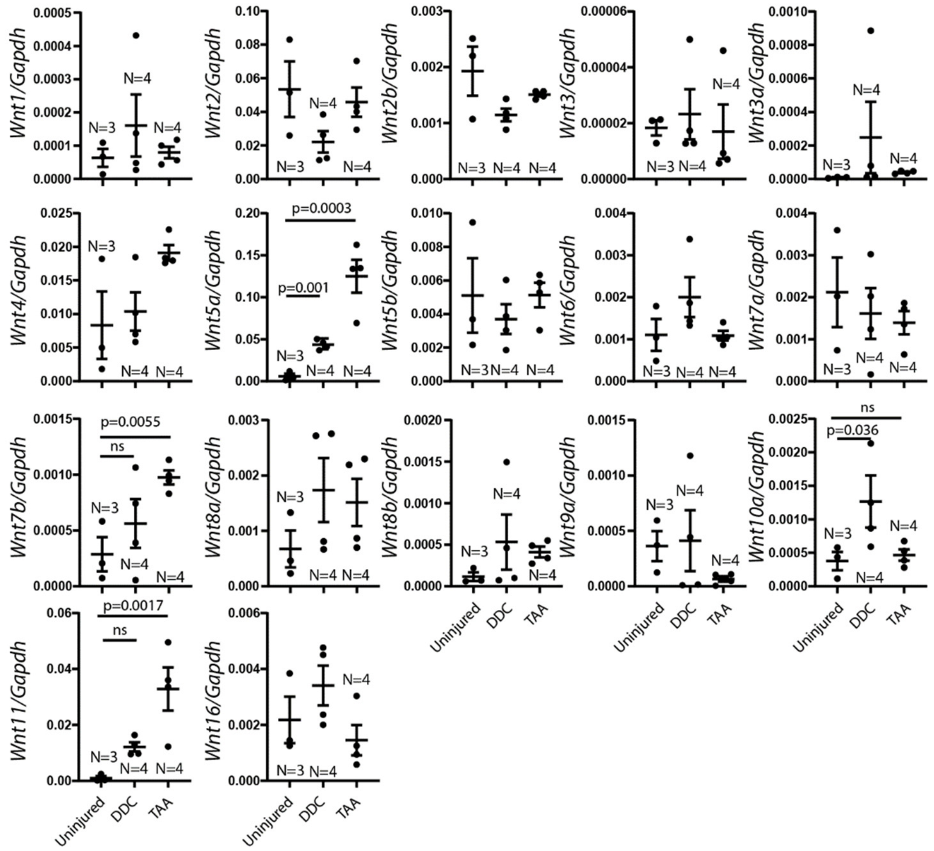

Supplementary Figure 2: a. mRNA expression of all detectable Wnt ligands in uninjured bile ducts, DDC injured and TAA injured. Source data are provided as a Source Data file. When comparing multiple groups, a One-way Anova with post-hoc correction for multiple testing is used. In dot-plots, data presented as mean ± S.E.M. Each data-point (N) represents an individual animal or patient.

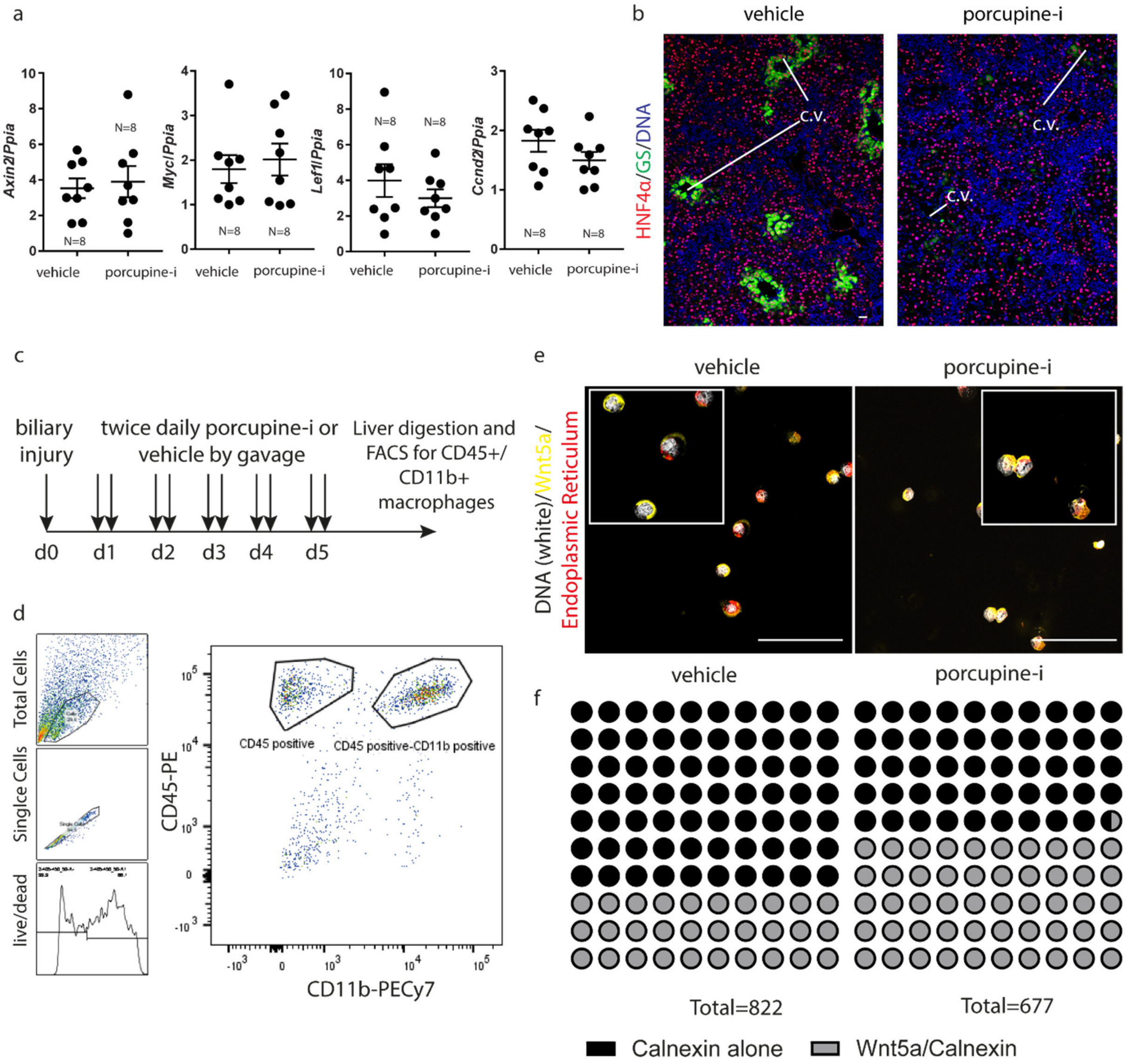

Supplementary 3: a. mRNA expression of β-catenin target genes in bile ducts isolated from mice with biliary disease treated with either Porcupine inhibitor (LGK974) or vehicle alone. **b.** Immunofluorescence of liver tissue treated with vehicle or Porcupine inhibitor, stained for Hnf4α (red) and Glutamine Synthase (GS, green). **c.** Schematic representing the Porcupine-i dosing regimen used to demonstrate that porcupine-I is effective in macrophages. **d**. Representative FACS plots showing the parameters by which CD45+/CD11b+ macrophages were isolated from livers undergoing biliary repair. **e.** Immunocytochemistry showing Wnt5a (yellow) and the endoplasmic reticulum (Calnexin, Red) in FACS isolated macrophages from mice treated with vehicle or the Porcupine inhibitor, LGK974. **f.** Quantification of macrophages in which WNT5A is found in the Endoplasmic Reticulum (Wnt5a/Calnexin dual positive cells). Scale bar = 50µm. c.v. denotes central vein. Source data are provided as a Source Data file. A Student's t-test is used to compare data. In dot-plots, data presented as mean ± S.E.M. Each data-point (N) represents an individual animal. In Supplementary 3f N=individual cells from three individual animals.

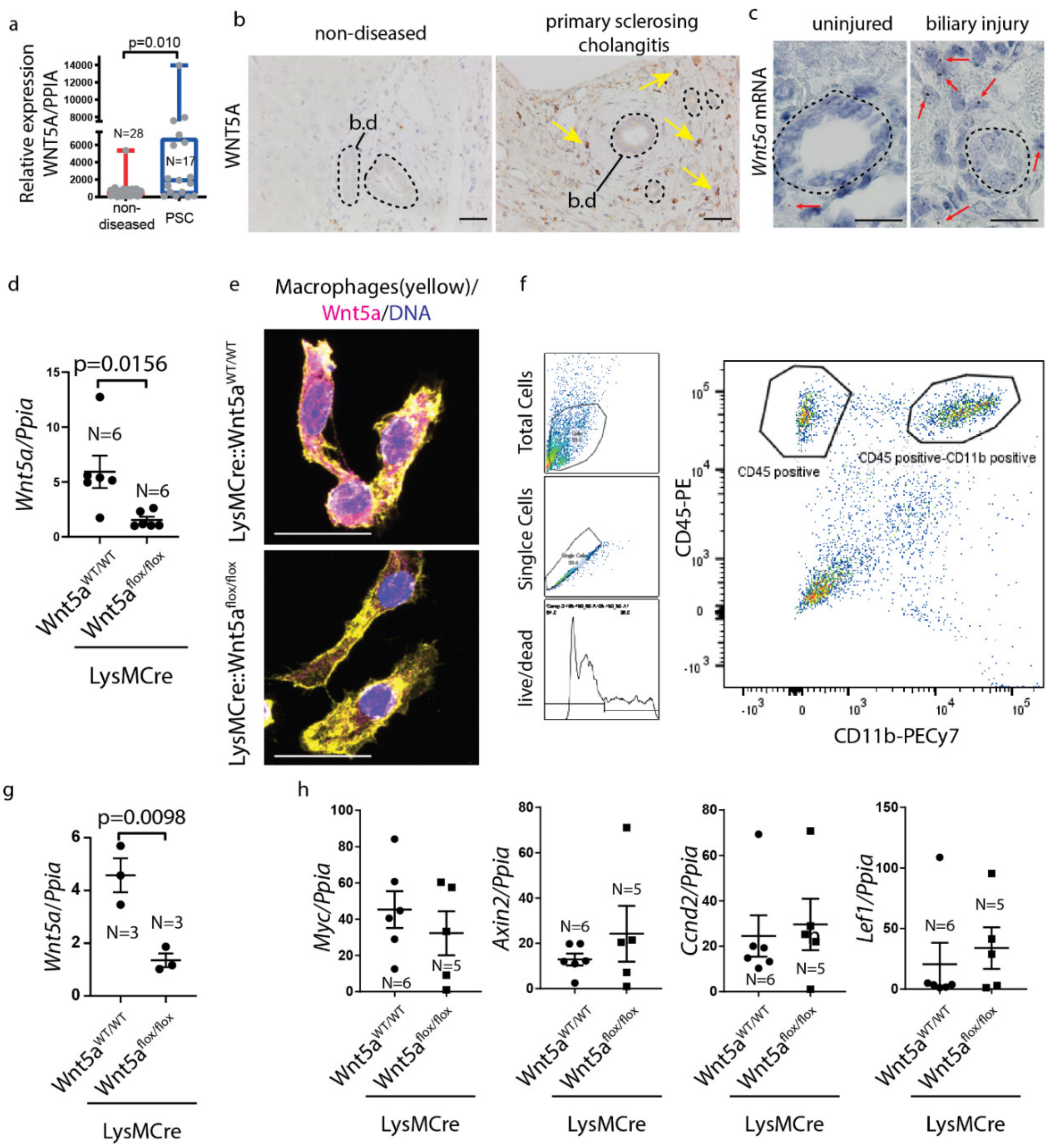

Supplementary Figure 4: a. mRNA expression of *Wnt5a* in non-diseased human tissue and tissue from patients with PSC. **b.** Immunohistochemistry of WNT5A in non-diseased liver and liver with PSC. Bile ducts are demarcated with a dotted line, yellow arrows denote positive cells. **c.** RNAScope for *Wnt5a* in bile ducts of uninjured mice and mice with biliary injury. Red arrows denote positive cells. **d.** mRNA expression of *Wnt5a* normalised to the housekeeping gene, *Ppia* In bonemarrow derived macrophages. **e.** Immunofluorescence of BMDMs (F4/80, yellow) from WT or Wnt5a$^{flox/flox}$ mice showing staining for WNT5A (magenta) and DNA (blue). **f.** Representative FACS plots showing gating strategy for isolating macrophages from livers undergoing biliary repair. **g.** mRNA expression of Wnt5a compared to housekeeping gene, *Ppia* in primary liver macrophages isolated from LysM::Wnt5a$^{flox/flox}$ mice or control. **h.** mRNA expression of β-catenin target genes in LysMCreWnt5a$^{WT/WT}$ versus in LysMCreWnt5$^{flox/flox}$ following biliary injury. Scale bars = 50µm. b.d. denotes bile duct. Source data are provided as a Source Data file. In graphs where two groups are included, a Student's t-test is used. Box-whisker plots represent min-max range of the data. In dot-plots, data presented as mean ± S.E.M. Each data-point (N) represents an individual animal or patient.

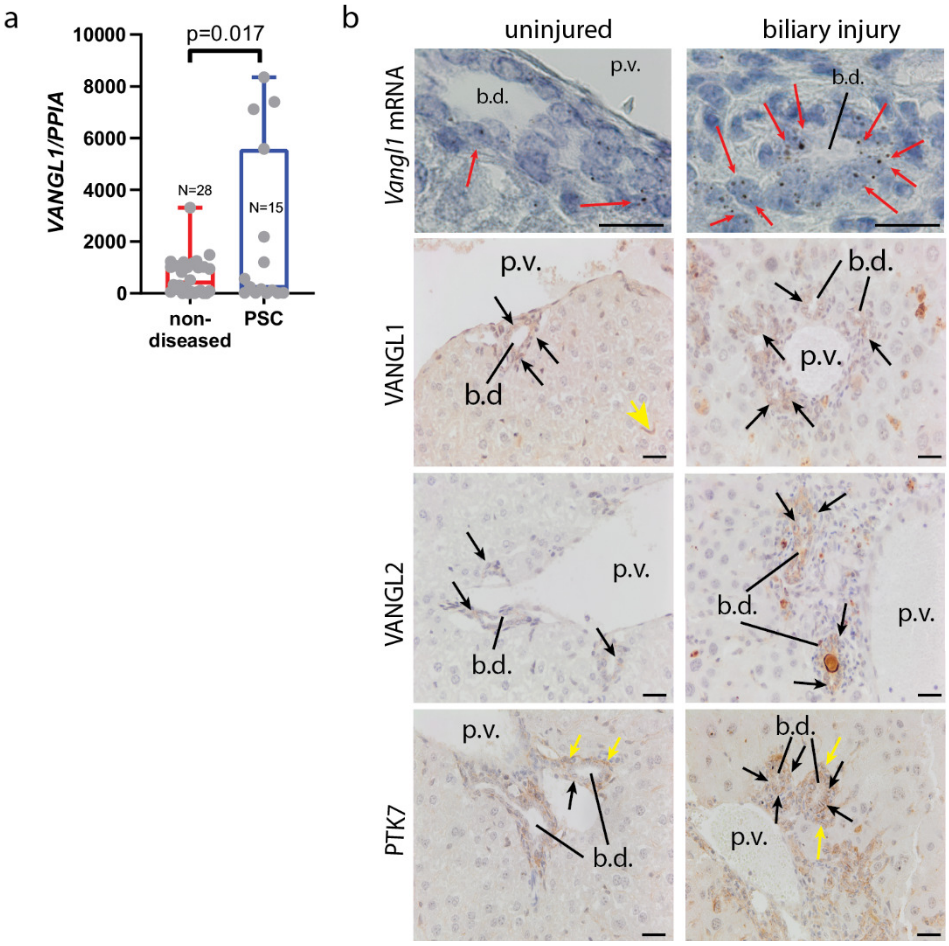

Supplementary Figure 5: a. mRNA expression of Vangl1 in non-diseased human liver and from patients with Primary Sclerosing Cholangitis. **b.** RNAScope (upper panels) for Vangl1 mRNA in uninjured mouse liver compared to mouse livers with biliary disease, red arrows denote positivity. Lower panels, immunohistochemistry for VANGL1, VANG2 and PTK7 in uninjured liver and livers from mice with biliary disease black arrows denote positivity within the bile duct. Yellow arrows denote positive wells outside of the bile duct. Scale bar = 50µm. b.d. denotes bile duct, p.v. denotes portal vein. Source data are provided as a Source Data file. In graphs where two groups are included, a Student's t-test is used. Box-whisker plots represent min-max range of the data. Each data-point (N) represents an individual animal or patient.

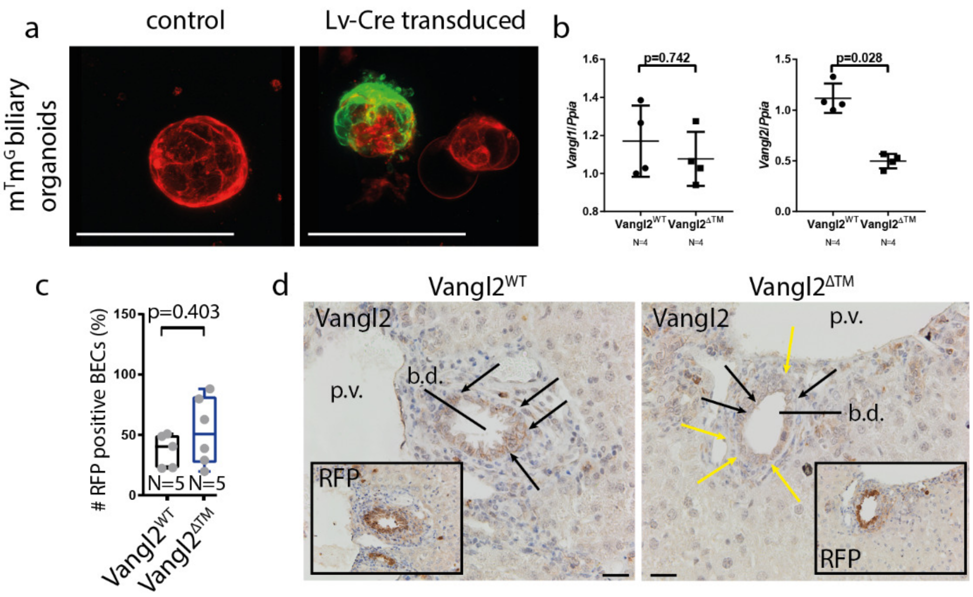

Supplementary Figure 6: a. mTmG organoids transduced with lentivirus carrying an antibiotic resistance cassette (left panel) or carrying Cre recombinase (right panel). Green fluorescence denotes cells in which Cre has recombined the loxP sites in the mTmG construct. **b**. mRNA expression of Vangl1 and Vangl2 in Vangl2^{WT} compared to Vangl2$^{\Delta TM}$ organoids. **c.** Quantification of RFP-positive (Cre-active) cells in Vangl2^{WT} and Vangl2$^{\Delta TM}$. **d.** Immunohistochemistry for VANGL2 in Vangl2^{WT} and Vangl2$^{\Delta TM}$ mice. Black arrows denote Vangl2 membrane positivity in cholangiocytes. Yellow arrows demarcate Vangl2-negative cholangiocytes. Inset, RFP staining, identifying the cholangiocytes that have activated Cre-recombinase. b.d. denotes bile ducts and p.v., portal vein. Scale bar = 50µm. Source data are provided as a Source Data file. In graphs where two groups are included, a Student's t-test is used. Box-whisker plots represent min-max range of the data. In dot-plots, data presented as mean ± S.E.M. In supplementary figure 6c each data-point (N) represents an individual animal. In supplementary figure 6b, each N represents an experimental replicate.

Supplementary References:

1. Kamimoto, K. *et al.* Heterogeneity and stochastic growth regulation of biliary epithelial cells dictate dynamic epithelial tissue remodeling. *Elife* (2016). doi:10.7554/eLife.15034

2. Hu, M. *et al.* Wnt/β-Catenin Signaling in Murine Hepatic Transit Amplifying Progenitor Cells. *Gastroenterology* (2007). doi:10.1053/j.gastro.2007.08.036

3. Planas-Paz, L. *et al.* YAP, but Not RSPO-LGR4/5, Signaling in Biliary Epithelial Cells Promotes a Ductular Reaction in Response to Liver Injury. *Cell Stem Cell* (2019). doi:10.1016/j.stem.2019.04.005

4. Okabe, H. *et al.* Wnt signaling regulates hepatobiliary repair following cholestatic liver injury in mice. *Hepatology* **64**, 1652–1666 (2016).

5. Itoh, T., Kamiya, Y., Okabe, M., Tanaka, M. & Miyajima, A. Inducible expression of Wnt genes during adult hepatic stem/progenitor cell response. *FEBS Lett.* (2009). doi:10.1016/j.febslet.2009.01.022

6. Wang, B., Zhao, L., Fish, M., Logan, C. Y. & Nusse, R. Self-renewing diploid Axin2 + cells fuel homeostatic renewal of the liver. *Nature* **524**, 180–185 (2015).

7. Planas-Paz, L. *et al.* The RSPO-LGR4/5-ZNRF3/RNF43 module controls liver zonation and size. *Nat. Cell Biol.* **18**, 467–479 (2016).

8. Tan, S. H. & Nusse, R. In vivo lineage tracing reveals Axin2-expressing, long-lived cortical thymic epithelial progenitors in the postnatal thymus. *PLoS One* (2017). doi:10.1371/journal.pone.0184582

9. Xing, L., Anbarchian, T., Tsai, J. M., Plant, G. W. & Nusse, R. Wnt/β-catenin signaling regulates ependymal cell development and adult homeostasis. *Proc. Natl. Acad. Sci.* (2018). doi:10.1073/pnas.1803297115

10. Nagaoka, T., Inutsuka, A., Begum, K., Musabbir Bin Hafiz, K. & Kishi, M. Vangl2 regulates E-Cadherin in epithelial cells. *Sci. Rep.* **4**, (2014).

11. Tward, A. D. *et al.* Distinct pathways of genomic progression to benign and malignant tumors of the liver. *Proc. Natl. Acad. Sci.* (2007). doi:10.1073/pnas.0706578104

12. Wiesner, S. M. *et al.* De novo induction of genetically engineered brain tumors in mice using plasmid DNA. *Cancer Res.* (2009). doi:10.1158/0008-5472.CAN-08-1800

13. Gay, D. M. *et al.* Loss of BCL9/9l suppresses Wnt driven tumourigenesis in models that recapitulate human cancer. *Nat. Commun.* (2019). doi:10.1038/s41467-019-08586-3

14. Lazaridis, K. N. & Larusso, N. F. The cholangiopathies. *Mayo Clinic Proceedings* **90**, 791–800 (2015).

15. Fickert, P. *et al.* A new xenobiotic-induced mouse model of sclerosing cholangitis and biliary fibrosis. *Am. J. Pathol.* **171**, 525–536 (2007).

16. Kim, Y. O., Popov, Y. & Schuppan, D. Optimized mouse models for liver fibrosis. in *Methods in Molecular Biology* **1559**, 279–296 (2017).

17. Burke, Z. D. *et al.* Liver Zonation Occurs Through a β-Catenin-Dependent, c-Myc-Independent Mechanism. *Gastroenterology* **136**, (2009).

18. Sampaziotis, F. *et al.* Reconstruction of the mouse extrahepatic biliary tree using primary human extrahepatic cholangiocyte organoids. *Nat. Med.* (2017). doi:10.1038/nm.4360
