# Supplementary material for: Non-canonical Wnt signalling regulates scarring in biliary disease via the planar cell polarity receptors
Source: Nat Commun. 2020 Jan 23;11:445. doi: 10.1038/s41467-020-14283-3 (PMC6978415; doi:10.1038/s41467-020-14283-3)
Supplement: Supplementary file 3 — Reporting Summary [file 41467_2020_14283_MOESM3_ESM.pdf]

nature research

Corresponding author(s): Dr Luke Boulter

Reporting Summary

Nature Research wishes to improve the reproducibility of the work that we publish. This form provides structure for consistency and transparency in reporting. For further information on Nature Research policies, see Authors & Referees and the Editorial Policy Checklist.

Statistical parameters

When statistical analyses are reported, confirm that the following items are present in the relevant location (e.g. figure legend, table legend, main text, or Methods section).

n/a	Confirmed	
☐	☒	The exact sample size (*n*) for each experimental group/condition, given as a discrete number and unit of measurement
☐	☒	An indication of whether measurements were taken from distinct samples or whether the same sample was measured repeatedly
☐	☒	The statistical test(s) used AND whether they are one- or two-sided<br>*Only common tests should be described solely by name; describe more complex techniques in the Methods section.*
☒	☐	A description of all covariates tested
☐	☒	A description of any assumptions or corrections, such as tests of normality and adjustment for multiple comparisons
☐	☒	A full description of the statistics including central tendency (e.g. means) or other basic estimates (e.g. regression coefficient) AND variation (e.g. standard deviation) or associated estimates of uncertainty (e.g. confidence intervals)
☒	☐	For null hypothesis testing, the test statistic (e.g. F, t, r) with confidence intervals, effect sizes, degrees of freedom and P value noted<br>*Give P values as exact values whenever suitable.*
☒	☐	For Bayesian analysis, information on the choice of priors and Markov chain Monte Carlo settings
☒	☐	For hierarchical and complex designs, identification of the appropriate level for tests and full reporting of outcomes
☒	☐	Estimates of effect sizes (e.g. Cohen's *d*, Pearson's *r*), indicating how they were calculated
☐	☒	Clearly defined error bars<br>*State explicitly what error bars represent (e.g. SD, SE, CI)*

Our web collection on statistics for biologists may be useful.

Software and code

Policy information about availability of computer code

Data collection	Imaging data was collected using NIS Elements for confocal images or cellSens for light microscopy. Quantitative PCR data was acquired using BD Lightcycler software v4.1
Data analysis	All data was analysed using FIJI, Microsoft Excel or Graph Pad Prism (version 6 or 7).

For manuscripts utilizing custom algorithms or software that are central to the research but not yet described in published literature, software must be made available to editors/reviewers upon request. We strongly encourage code deposition in a community repository (e.g. GitHub). See the Nature Research guidelines for submitting code & software for further information.

Data

Policy information about availability of data

All manuscripts must include a data availability statement. This statement should provide the following information, where applicable:

- Accession codes, unique identifiers, or web links for publicly available datasets
- A list of figures that have associated raw data
- A description of any restrictions on data availability

The primary data that support the findings of this study are available from the corresponding author upon request. All data included in the figures is avaliable in the source data file associated with this manuscript

Field-specific reporting

Please select the best fit for your research. If you are not sure, read the appropriate sections before making your selection.

☒ Life sciences ☐ Behavioural & social sciences

For a reference copy of the document with all sections, see nature.com/authors/policies/ReportingSummary-flat.pdf

Life sciences

Study design

All studies must disclose on these points even when the disclosure is negative.

Sample size	Number of mice in experimental groups was calculated based on mice having 100% penetrance of injury and 60% change in effect (based on previous intervention studies in this model) with a variation of 20%, thus requiring 4 animals per group assuming a power of 80% and alpha value of 0.05. Therefore we have ensured that there are a minimum of 4 mice per group.
Data exclusions	No data was excluded from the analysis.
Replication	For in vitro experiments all data-points represent a biological replicate and there were no data which did not replicate. In vivo, all data points represent discrete biological replicate, there was no re-sampling of animals and therefore each data point represents an N=1, total N=values are given throughout.
Randomization	In all animal studies animals were randomized into groups before the study. Should animals be receiving a common, initial treatment such as Tamoxifen administration, they are re-randomized following this into treatment groups.
Blinding	Analysis of all tissue including histological quantification was performed blinded to prevent user bias.

Materials & experimental systems

Policy information about availability of materials

n/a	Involved in the study
☐	☒ Unique materials
☐	☒ Antibodies
☐	☒ Eukaryotic cell lines
☐	☒ Research animals
☐	☒ Human research participants

Unique materials

Obtaining unique materials	All unique material is available to other researchers.

Antibodies

Antibodies used	All antibodies, antigen retrieval and concentrations are described in this study are summerised in table-2 in materials and methods
Validation	All antibodies were either pre-validated by the supplier (in the majority of cases) or validated in house using appropriate control tissues (known expressing tissues as a positive control and isotype primary antibodies as a negative).

Eukaryotic cell lines

Policy information about cell lines

Cell line source(s)	Organoid lines were derived from mutant mice and are available from the authors. Normal Human Cholangiocytes (NHC3 cells) were provided by Dr Jesus Banales (Spain) and listed as an author.
Authentication	Organoids were derived in house and have been tested for biliary marker expression by immunocytochemistry and qRT-PCR.
Mycoplasma contamination	Organoids and cell lines were intermittently tested for mycoplasma contamination in house and were found to be negative
Commonly misidentified lines (See ICLAC register)	No misidentified lines were used in this study

Research animals

Policy information about studies involving animals; ARRIVE guidelines recommended for reporting animal research

Animals/animal-derived materials	Mice were used throughout these studies and they are detailed within the materials and methods. All mice used experimentally are male, 6-8 weeks old. Mice not derived from a transgenic or mutant colony and Vangl2GFP mice are on an inbred CD1 background. LysMCreWnt5a and K19Vangl2flox lines are C57Bl6/J and Vangl2S464N mice are C3H. Throughout, control mice from transgenic colony are derived from breeding and lack either the mutation or the flox allele, thus maintaining the background genetics between experimental and control mice.

Human research participants

Policy information about studies involving human research participants

Population characteristics	Two populations of patients were used in this study. Non-injured liver was acquired from the sudden-death brain bank, Edinburgh, UK. The second population of patients was identified by an NHS pathologist from the ACCORD database, NHS Lothian and all had pathologically confirmed Primary Sclerosing Cholangitis.

Method-specific reporting

n/a	Involved in the study
☒	☐ ChIP-seq
☐	☒ Flow cytometry
☒	☐ Magnetic resonance imaging

Flow Cytometry

Plots

Confirm that:

☒ The axis labels state the marker and fluorochrome used (e.g. CD4-FITC).

☒ The axis scales are clearly visible. Include numbers along axes only for bottom left plot of group (a 'group' is an analysis of identical markers).

☒ All plots are contour plots with outliers or pseudocolor plots.

☒ A numerical value for number of cells or percentage (with statistics) is provided.

Methodology

Sample preparation	Whole livers were isolated from LysMCre::Wnt5a flox mice following liver injury. Livers were digested using a combination of collagenase and dnase to remove hepatocytes, leaving parenchymal cells. These cells were stained for CD45-PE and CD11B-PECy7 (macrophages). Live cells were idnetified with DAPI and were sorted based on DAPI-/CD45+/CD11B+
Instrument	BD LSRFortessa
Software	Flow cytometry acquisition BD FACSDiva v6.1 software was used. FlowJo was used to analyse data
Cell population abundance	Cells were highly pure (although this was not formally assessed) but were stained for the macrophage marker F4/80.
Gating strategy	Cells were identified by their SSC-A against FSC-A. We gated for events of a suitable size and granularity, thereby excluding debris and cell aggregates. These cells were assessed using FSC-A against FSC-H to define single cell events and exclude cells that were in small clusters or duplicates. Single cells were analysed for H2B-GFP positivity using 488-525_50-A lasers and filters in combination.

☒ Tick this box to confirm that a figure exemplifying the gating strategy is provided in the Supplementary Information.
